# Mechanisms underlying antidepressant effect of transcutaneous auricular vagus nerve stimulation on CUMS model rats based on hippocampal α7nAchR/NF-κB signal pathway

**DOI:** 10.1186/s12974-021-02341-6

**Published:** 2021-12-17

**Authors:** Jun-ying Wang, Yue Zhang, Yu Chen, Yu Wang, Shao-yuan Li, Yi-fei Wang, Zi-xuan Zhang, Jinling Zhang, Peijing Rong

**Affiliations:** Department of Physiology, Institute of Acupuncture and Moxibustion, China Academy of Chinese Medical Sciences, Dongcheng District, No.16 Dongzhimen Nan Xiao Street, Beijing, 100700 China

**Keywords:** Depression, Transcutaneous auricular vagus nerve stimulation, Neuroinflammation, Alpha 7 nicotinic acetylcholine receptor, Cytokines

## Abstract

**Background:**

Stress-induced neuroinflammation was considered to play a critical role in the pathogenesis of depression. Transcutaneous auricular vagus nerve stimulation (taVNS) is a relatively non-invasive alternative treatment for patients suffering from major depressive disorder. The anti-inflammatory signal of vagus nerve is mediated by α7 nicotinic acetylcholine receptor (α7nAchR), and the hippocampus, the region with the most distribution of α7nAchR, regulates emotions. Here, we investigated the role of α7nAchR mediating hippocampal neuroinflammation in taVNS antidepressant effect though homozygous α7nAChR (−/−) gene knockout and α7nAchR antagonist (methyllycaconitine, MLA).

**Methods:**

There were control, model, taVNS, α7nAChR(−/−) + taVNS, hippocampus (Hi) MLA + taVNS and Hi saline + taVNS groups. We used the chronic unpredicted mild stress (CUMS) method to establish depressive model rats for 42 days, excepting control group. After the successful modeling, except the control and model, the rats in the other groups were given taVNS, which was applied through an electroacupuncture apparatus at the auricular concha (2/15 Hz, 2 mA, 30 min/days) for 21 days. Behavioral tests were conducted at baseline, after modeling and after taVNS intervention, including sucrose preference test (SPT), open field test (OFT) and forced swimming test (FST). These tests are widely used to evaluate depression-like behavior in rats. The samples were taken after experiment, the expressions of α7nAchR, NF-κB p65, IL-1β and the morphology of microglia were detected.

**Results:**

Depression-like behavior and hippocampal neuroinflammation in CUMS model rats were manifested by down-regulated expression of α7nAchR, up-regulated expression of NF-κB p65 and IL-1β, and the morphology of microglia was in amoebic-like activated state. TaVNS could significantly reverse the above-mentioned phenomena, but had rare improvement effect for α7nAChR(−/−) rats and Hi MLA rats.

**Conclusion:**

The antidepressant effect of taVNS is related to hippocampal α7nAchR/NF-κB signal pathway.

## Background

Major depressive disorder (MDD) is a highly prevalent mental health condition characterized by low mood, anhedonia, reduced energy, ruminative thoughts, impaired cognition, vegetative symptoms and suicidal attempts [1]. Epidemiological studies showed that the total estimated number of patients with depression worldwide is about 350 million, which is a leading cause of disability worldwide with a global prevalence of 2.6–5.9% [2]. According to global projections, MDD will be the single major cause of the burden of all health conditions by 2030 [3]. Despite of more and more available pharmacotherapeutic options available, it is estimated that about 30–60% of patients fail to respond to available treatments [4], and are associated with gastrointestinal reactions, liver toxicity and other side effects [5]. While about 40% of patients have residual symptoms after drug withdrawal and the recurrence rate for depression is 85% within 10 years [6]. Therefore, it is critical to develop more effective non-pharmaceutical treatment options including bioelectronic medicine.

VN is the 10th cranial nerve, which is mainly a sensory nerve and essentially relays biofeedback to the brain [7]. In 2005, the US Food and Drug Administration approved vagus nerve stimulation (VNS) for refractory MDD patients [8]. Significantly, VNS produces a systemic anti-inflammatory effect which may be an important reason for its effectiveness in patients who do not respond to anti-depressants [9]. However, VNS requires surgery, and is limited by postoperative complications, such as dyspnea, pharyngitis and so on [10]. Researches from neuroanatomy show that the only branch of vagus nerve in the body surface is auricular branch of the vagus nerve (ABVN) [11], which mainly distributes in the external auditory meatus and concha (cymba conchae and cavum conchae), and the cymba conchae is supplied exclusively by the ABVN [12]. The transcutaneous auricular vagus nerve stimulation (taVNS), as one of the promising electrotherapies, has been used in the treatment of depression [13]. Clinical studies show that taVNS can greatly improve the scores of Hamilton Depression Rating Scale (HAMD) and self-rated depression scale in patients with depression without surgery, and produce clinical efficacy similar to that of VNS [10, 14]. taVNS is frequently used in the treatment of MDD, especially for residual symptoms [15]. Previous studies by our team have shown that the antidepressant effect of taVNS is mediated by the default network of nucleus tractus solitarius–limbic lobe–brain, and the hippocampus is an important part of the limbic lobe, and the abnormalities of hippocampus are related to depression causality [16]. But its potential molecular mechanism in the brain is not completely elucidated.

A large number of evidences suggest that there is a close link between neuroinflammation and depression [17], and stress-induced neuroinflammation characterized by overproduction of inflammatory cytokines in the brain acts as an important pathogenesis of depression [10]. The preclinical studies have also shown that chronic stress can lead to the activation of the innate immune system in the brain, while the increase of inflammatory cytokines, such as IL-1β can lead to depressive-like behavior in animals [18]. Thus, it can be inferred that the anti-inflammatory therapy may be a promising novel option [19].

Studies have shown that VN can serve as a bridge between the central nervous system (CNS) and the immune system, playing an important role in regulating inflammation [20]. Our previous results suggest that taVNS significantly improved depression-like behavior and pain intensity as well as decreased the release of TNF-α in plasma, hypothalamus and hippocampus of the CUMS with chronic constriction injury of the sciatic nerve (CCI) model rats [21]. Additionally, we found that taVNS could not suppress lipopolysaccharide-induced TNF-α and NF-κB p65 after vagotomy or with αlpha-7 nicotinic acetylcholine receptors (α7nAChRs) antagonist injection, which demonstrated that taVNS could be utilized to improve lipopolysaccharide-induced inflammatory responses via α7nAChR-mediated cholinergic anti-inflammatory pathway (CAP) [22].

α7nAChR, an ionotropic receptor mainly expressed in the CNS microglia, is thought to play a role in a wide range of psychiatric and neurological disorders and is activated by cellular danger signals such as NF-κB [23, 24]. TaVNS serves an important role in the relationship among the spleen, gut, brain and inflammation. α7nAChR mediates vagus nerve anti-inflammatory signals and is involved in regulating central cholinergic pathways of microglial activation [25]. Furthermore, hippocampus is an important site of α7nAChR in regulating behavioral or emotional response to stress in CUMS rats [26]. Accordingly, here we sought to understand whether taVNS could improve behavioral deficits and decrease neuroinflammation in rats exposed to CUMS, and whether this anti-inflammatory-based antidepressant effect is mediated by the regulation of α7nAChR. Specifically, we examined behaviors at time points, including anhedonia, exploration, and motor activity, and detected α7nAchR and related neuroinflammatory markers (microglia, NF-κB p65, phosphorylated NF-κB p65, IL-1β) in the hippocampus at harvest by using homozygous α7nAChR(−/−) gene knockout rats.

## Methods

### Animal care

Male Sprague–Dawley (SD) rats, 6 weeks old (200 ± 20 g, *n* = 60) were obtained from the Laboratory Animal Center of Academy of Military Medical Sciences (Beijing), and homozygous α7nAChR(−/−) gene knockout rats, 6 weeks old (200 ± 20 g, *n* = 10) were obtained from Cyagen Biosciences (Guangzhou). All rats were housed in a controlled environment of 20–25 °C, with humidity of 55 ± 2%, and in quiet states maintained under a 12 h/12 h light/dark cycles with ad libitum access to food and water (except when indicated). All procedures were reviewed and approved by the Institute of Animal Care and Use Committee of Institute of Acupuncture and Moxibustion, China Academy of Chinese Medical Sciences (Permit No. D2019-02-11-3). Also, all animal experiments complied with the ARRIVE guidelines and were carried out according to the National Institutes of Health guide for the care and use of laboratory animals. Due to protection of estrogen on stress, only male rats were chosen for this study. The rats exposed to CUMS were housed separately in different cages for social isolation and five rats per cage were housed in the control group.

### Experimental design

**Fig. 1:**
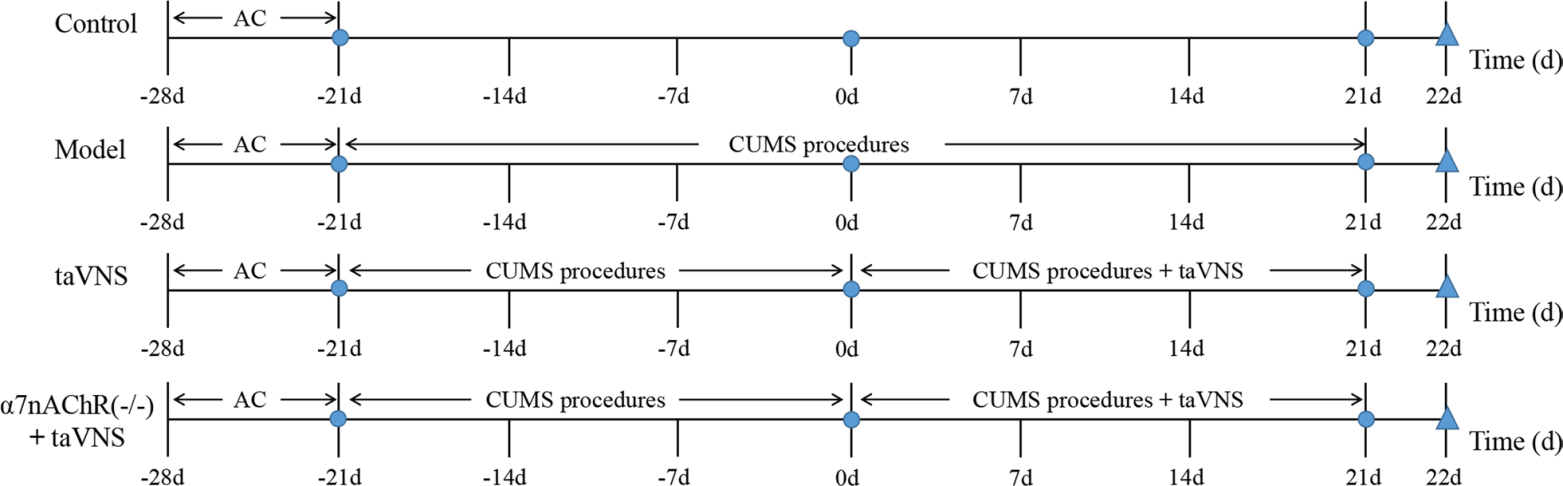
Experimental procedure. AC, acclimatization; CUMS, chronic unpredictable mild stress; Hi, hippocampus; taVNS, transcutaneous auricular vagus nerve stimulation; − 21 days, 0 day, 21 days involved three behavioral testing of SPT, OFT and FST

**Fig. 2:**
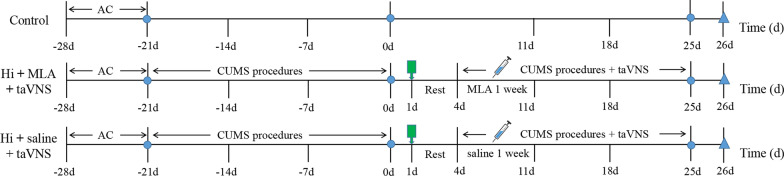
Experimental procedure. Embedding cannulae at 1 day; make samples on the last day. − 21 days, 0 day and 25 days involved three behavioral testing of SPT, OFT and FST

After adaptive feeding for 7 days, SD rats were randomly assigned into control group, model group, taVNS group, Hi MLA plus taVNS group and Hi saline plus taVNS group. The modeling and intervention methods among taVNS group, α7nAChR(−/−) plus taVNS group, Hi α7nAChR antagonist plus taVNS group and Hi saline plus taVNS group was the same. There were 10 rats in each group. During the modeling period, all groups, excepted control group, were subjected to social isolation and CUMS for 42 days. Since the modeling successfully, the dorsal hippocampus was embedded the cannulae in Hi MLA plus taVNS group and Hi saline plus taVNS group. Rest for 3 days after embedding the cannulae, two groups rats were infused MLA or saline 80 μg/kg each time with 1-week continuous unilaterally local infusions. 1 h before CUMS procedure, rats in the taVNS group, α7nAChR(−/−) plus taVNS group, Hi MLA plus taVNS group and Hi saline plus taVNS group were administered with taVNS once daily for 21 days. Sucrose preference test (SPT), open field test (OFT), and force swimming test (FST) were carried out on all rats to ensure the consistency of baseline characteristics before the implementation of the experimental guidelines. The detailed experimental procedure is shown in Figs. 1, 2.

### Preparation of the CUMS model

The CUMS model has been validated as one of the most relevant rodent models of depression [27]. In this study, the CUMS model was modified according to the methods previously described [28], and some appropriate adjustments were made to enhance unpredictability. Rats were subjected to 7 different stressors: clip tail for 3 min, swimming in cold water (4 ℃, 5 min), food deprivation for 24 h, housing in a wet cage (24 h, 200 ml of water mixed with 100 g of sawdust), water deprivation (24 h), continuous overnight illumination (12 h) and electric shock feet (1 mA, 10 s each time, 10 s interval, 2 min) [29]. One of these stressors were performed every day in a random order for rats, and each stressor would not be arranged 2 days in a row to avoid a rat predicting it.

### Intervention of taVNS

**Fig. 3:**
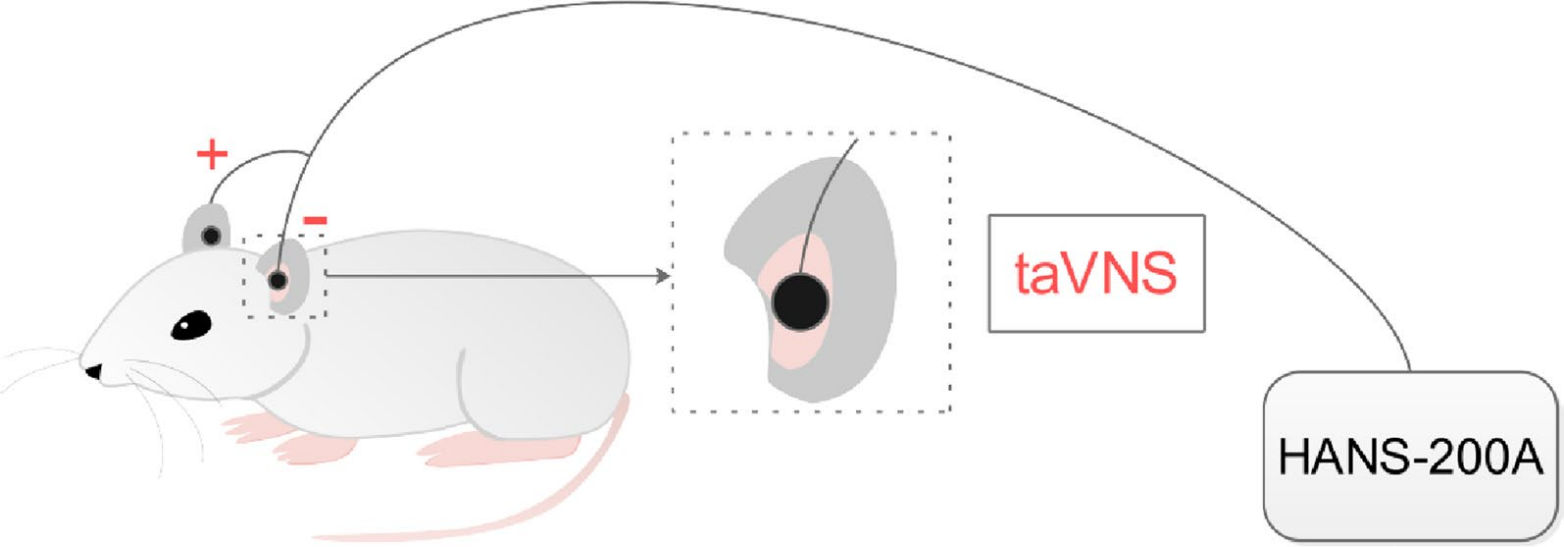
Intervention of taVNS. The skin receptive area of taVNS is located in the auricular concha with ABVN distribution

On the 21st day of modeling, the SPT, OFT and FST were used to observe whether the model of CUMS rats was established successfully. On the basis of successfully built model, taVNS group, α7nAChR(−/−) plus taVNS group, Hi MLA plus taVNS group and Hi saline plus taVNS group were administrated taVNS for consecutive 21 consecutive days. Electroacupuncture apparatus (HANS-200A, Nanjing Jisheng Medical Technology Co., Ltd.) was used for stimulation. During the intervention, the rats were anesthetized continuously with 2% isoflurane inhalant (Hebei Nine Sent Pharmaceutical Co., Ltd., Hebei, China) and then the two opposite magnetic electrodes (±) and a homemade metal ear splint (2 cm length, 0.5 cm width, 0.05 cm thickness) were merged and connected to the auricular concha to form an electron circle [30] (see Fig. 3). The stimulation parameters are as follows: ① stimulation frequency: 2/15 Hz (2 and 15 Hz, switched every second); ② stimulus intensity: 2 mA; ③ stimulation duration: 30 min per day [21].

### Body mass

In this study, the body mass of rats was weighed before modeling, after modeling and after intervention, respectively. Body mass, an important indicator for judging whether the rat model is successful and whether the intervention is effective, can be used to mimic somatic symptoms of patients with depression, such as changes in appetite [31].

### Intracerebroventricular infusion

On the 21st day of modeling, intracerebroventricular infusion was performed in the Hi α7nAChR antagonist + taVNS and Hi saline + taVNS groups. Twenty rats were anaesthetized by inhalation of 2% isofluorane (Hebei Nine Sent Pharmaceutical Co., Ltd., Hebei, China), which were cannulated in the dorsal hippocampus. A longitudinal incision was made over the scalp, and then, the skull was exposed. The incisor bar adjusted such that bregma and lambda were at the same height. The dorsal hippocampus coordinates were positioned at 3.6 mm posterior to bregma, 2.75 mm lateral, and 3.0 mm vertical (according to the atlas of Paxinos and Watson). Three screws were anchored in the skull between the cannulae; wire was wrapped around the screws. These were used to help anchor the protective cap that was formed by cranioplastic cement, which was applied to surround and secure the cannulae. The rats were given 3 days of rest after surgery. The cannulae (RWD Co., Ltd., Shenzhen, China) fixed on a polyethylene tube, and connected to a syringe microinjector (50 μl, Hamilton) [32]. α7nAchR antagonist methyllycaconitine (MLA, MedChemExpress Co., Ltd., China) dissolved in sterile saline (2 mg/ml, 2.29 mM). Referred and adjusted the dose of MLA in literature [33], the infusion procedure was programmed at the rate of 1 μl/min and delivered by a micro syringe pump. The MLA was infused at a dose of 80 μg/kg each time with 1-week continuous unilaterally local infusions. After each infusion, the injector cannula was remained in the guide cannula for 10 min. Same volume of sterile saline was infused as control. The same procedures and same volume of sterile saline was infused to the Hi saline group.

### Behavioral testing

#### Sucrose preference test (SPT)

SPT, as described before, was performed before modeling, after modeling and after intervention, respectively. Anhedonia is considered to be one of the core symptoms of depressive disorder, and the condition of anhedonic-like behaviors of rats in the study were evaluated by SPT [34]. Rats were trained to adapt to 1% sucrose solution (Amresco 0335, USA) during the adaptation cycles. After the adaptation, all rats were deprived of food and water for 23 h. Then they rats were all housed in individual cages and had free access to two pre-weighed bottles containing 240 ml sucrose solution (1% w/v) and 240 ml pure water for 1 h. At the end of the test, the bottles of 1% sucrose solution and pure water were re-weighted and recorded. The percentage difference in sucrose preference was calculated by the following formula: sucrose preference ratio (%) = sucrose consumption/(sucrose consumption + pure water consumption) × 100 [35].

### Open field test (OFT)

OFT of rats was carried out before modeling, after modeling and after intervention, respectively, which is commonly used to measure general locomotor activity and willingness to explore in rodents [36, 37]. The apparatus used is a square arena, which is made of an 100 cm × 100 cm × 40 cm plastic board without special smell, characterized by a black wall and a black base, and the base is divided into equal squares of 20 cm × 20 cm by white stripes. When the rats were gently placed in the center of the square arena, they were allowed to enjoy autonomous movement and free exploration, while we monitored and recorded the scores of horizontal movement (at least three paws getting into the same square once counted as 1 point) and the scores of vertical movement (the rat standing upright on its hind legs once counted as 1 point) in 3 min. The OFT apparatus was cleaned with 75% ethanol after each rat was tested to avoid the interference of the odor signal left by this rat to the next one. The whole experiment process was recorded by a camera about 120 cm above the apparatus.

### Force swimming test (FST)

In this study, FST of rats was done before modeling, after modeling and after intervention, respectively, which is commonly used to evaluate the level of desperation in the rodents behavior [38]. Before the experiment, the rats were individually placed in a forced swimming bucket (a white plastic cylinder, height of 60 cm, diameter of 30 cm, water depth of 45 cm). The rats swam in water at 24–26 °C for 15 min one day before the experiment began to adapt to the environment. On the day of the experiment, each rat swam for 5 min, and a video camera was set up above the bucket to observe and record the immobility time of the rats clearly. Remove 1 min before and after the FST, and count the 3-min immobility time. The immobility was defined when a rat no longer struggled or just floated in the water, excluding subtle limb movements to keep breathing [39].

### Western blot analysis

After completion of the experimental procedure, the rats were anesthetized with an injection of pentobarbital sodium (35 mg/kg body weight, i.p.) followed by decapitation. The hippocampus samples were collected on ice. After washed with pre-chilled sterile saline solution, hippocampus samples were stored in pre-chilled 1.5 ml cryogenic microtube and experienced a snap frozen in liquid nitrogen. Samples were then placed in a freezer at -80 °C for futher experimental procedures. The samples were homogenized in RIPA lysis buffer with protease inhibitor cocktail for protein extraction. Then the supernatant was collected and centrifuged at 13,000 rpm and 4 °C for 20 min. The protein concentration was determined by the bicinchoninic acid (BCA) method. Same amount of protein samples was separated in 5%, 12% or 15% SDS gels and transferred to PVDF membrane (0.2 or 0.45 μm). The membranes were blocked in 5% bull serum albumin (BSA) Tris-buffered saline plus Tween (BSA-TBST) for 1 h at room temperature (RT) and incubated at 4 °C overnight with the following primary antibodies: anti-NF-KB p65 (1:1000, Rabbit mAb, CST/8242); anti-Phospho NF-κB p65 (1:500, Rabbit mAb, CST/3033); anti-IL-1β (1:500, Rabbit polyclonal, Abcam/ab9722); anti-α7nAchR (1:500, Rabbit polyclonal, Abcam/ab10096). Equal loading was confirmed with anti-β-actin (1:3000, Mouse monoclonal, Abcam/ab6276). After the blots were washed in TBST for 5 times, the secondary antibodies (1:5000, respectively; Goat Anti-Mouse, Abcam/ab6789; Goat Anti-Rabbit, Abcam/ab6721) were incubated for 1 h at RT. The signal was captured on an ImageQuant LAS4000 mini image analyzer (GE Healthcare, Buckinghamshire, UK), and the band levels were quantified with Quantity One software v.4.6.2 (Bio-Rad, Hercules, CA, USA).

### Immunofluorescent staining analysis

Rats (*n* = 3 in each group) were perfused with saline, followed by 4% paraformaldehyde solution. The brain tissue was harvested and immersed in 25% sucrose solution (0.1 M PBS as solvent) until the tissue was sunken to the bottom of the tube. Each sample was cut into a 30 μm thick section (Microm International FSE, Thermo, USA). The free-floating sections were incubated in 0.01 M phosphate-buffer saline (PBS), washed with PBS Tween-20 (PBST) for three times and blocked with 5% normal donkey serum in 0.01 M PBST for 30 min at RT. For observation of the co-expression of α7nAchR, IL-1β, and ionized calcium-binding adapter molecule 1 (Iba-1), the sections were incubated in primary antibodies anti-Iba-1 (Goat polyclonal, 1:200, Abcam/ab5076), anti-α7nAchR (Rabbit polyclonal, 1:50, Abcam/ab10096) and anti-IL-1β (Rabbit polyclonal, 1:50, Abcam/ab9722) at 4 °C overnight. After PBS washing, the sections were incubated in a solution containing Alexa Fluor® 488-conjugated donkey anti-rabbit IgG (1:300, Abcam/ab150073), Cy3® preadsorbed-conjugated donkey anti-goat IgG (1:300, Abcam/ab6949) at RT for 2 h. The tissues were mounted in mounting medium containing DAPI (Genepool/GPB18242). The images were captured with the Olympus FV9100 system.

### Statistical analysis

Statistical analysis was conducted by SPSS 26.0 software package (IBM, Armonk, New York, NY, USA). All data were expressed as mean ± SD in the experiments of body mass, behavioral testing and western blot, which were analyzed by one-way ANOVA for repeated measurements. Once F ratios were significant, post hoc comparisons were made with the Tukey post hoc test. Differences between individual means were tested for significance according to Fisher’s least significant difference procedure. *P* < 0.05 was considered statistically significant.

## Results

### Body mass and behavioral experiments

#### Body mass

**Fig. 4:**
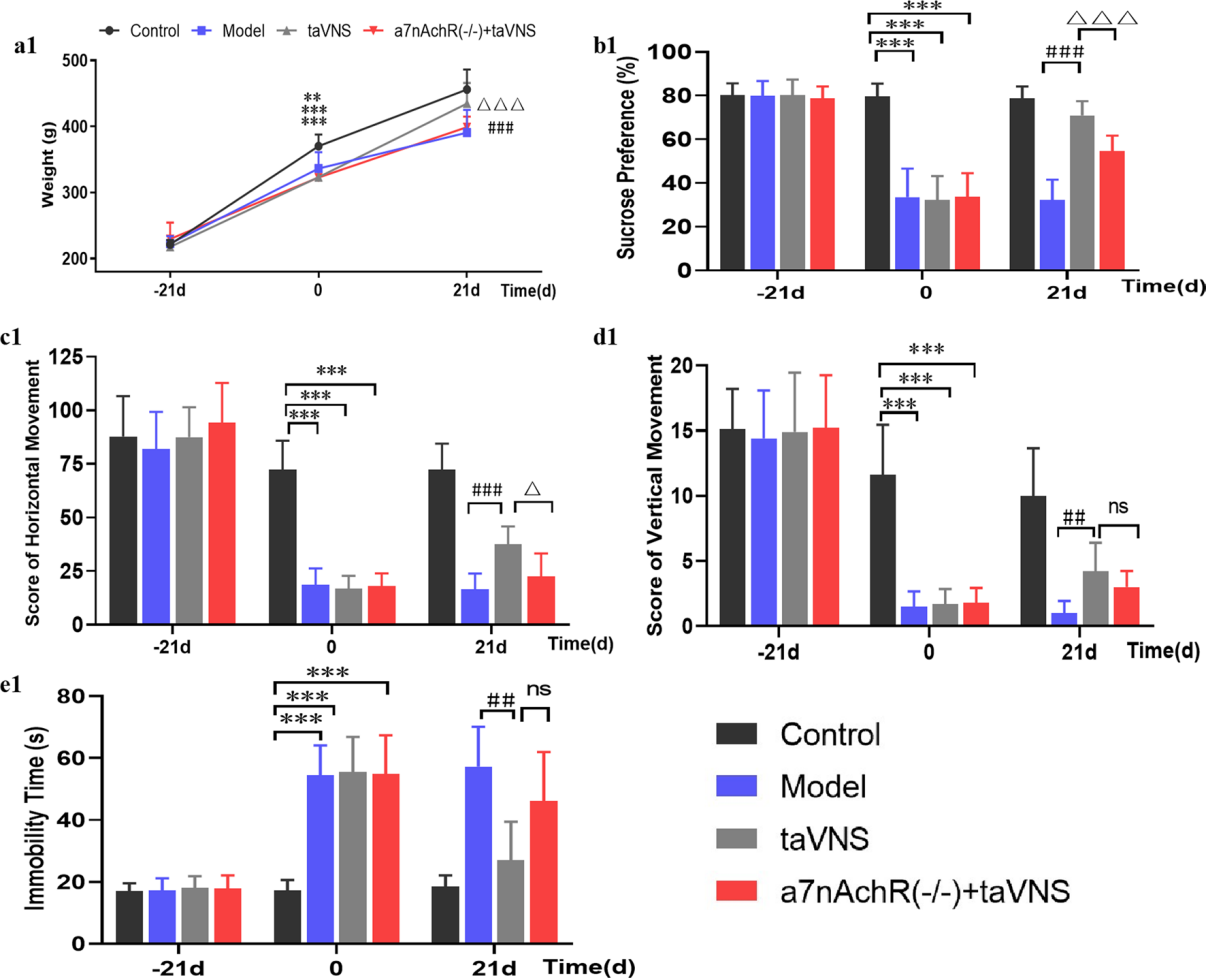
The effect of taVNS on the body mass and behavioral experiments of rats at different time points. **a1** Comparison of body weight of rats at different time points. **b1** Comparison of SPT of rats at different time points. **c1** Comparison of horizontal movement scores of rats in OFT at different time points. **d1** Comparison of vertical movement scores of rats in OFT at different time points. **e1** Comparison of immobility time of rats in FST at different time points. The values are presented as mean ± SD. One-way ANOVA with Tukey post hoc test was performed for statistical analysis. **P < 0.01 vs. the control group, ***P < 0.001 vs. the control group; ##*P* < 0.01 vs. the model group, ###*P* < 0.001 vs. the model group; △*P* < 0.05 vs. the taVNS group, △△△*P* < 0.001 vs. the taVNS group; ns: no significance (*n* = 10 per group)

At baseline (− 21 days), there was no significant difference in weight among groups (*P* > 0.05). At 0 day, the weight in model, taVNS and α7nAchR(−/−) + taVNS groups were significantly lower than that in the control group (*P* < 0.01, *P* < 0.001, *P* < 0.001). At 21 days, compared with model group, the weight in taVNS group significantly increased (*P* < 0.001). Compared with taVNS group, the weight in α7nAchR(−/−) + taVNS group markedly decreased (*P* < 0.001) (as shown in Fig. 4a1).

**Fig. 5:**
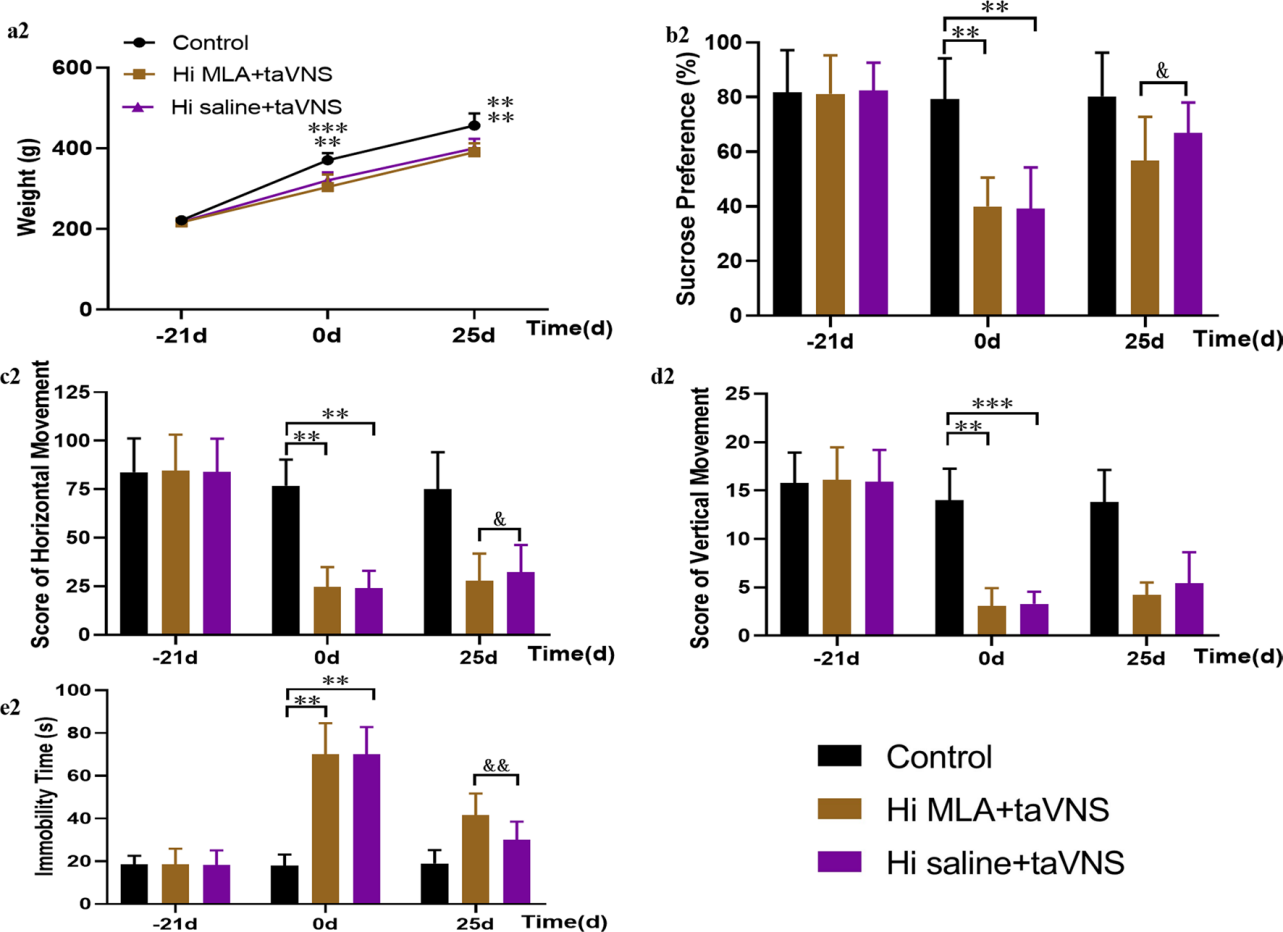
The effect of α7nAchR in the hippocampus on the body mass and behavioral experiments of CUMS rats intervened by taVNS at different time points. **a2** Comparison of body weight of rats at different time points. (**b2**) Comparison of SPT of rats at different time points. **c2** Comparison of horizontal movement scores of rats in OFT at different time points. **d2** Comparison of vertical movement scores of rats in OFT at different time points. **e2** Comparison of immobility time of rats in FST at different time points. The values are presented as mean ± SD. One-way ANOVA with Tukey post hoc test was performed for statistical analysis. **P < 0.01 vs. the control group, ***P < 0.001 vs. the control group; and *P* < 0.05 vs. the Hi MLA + taVNS group, &&*P* < 0.01 vs. the Hi MLA + taVNS group (*n* = 10 per group)

At baseline (− 21 days), there was no significant difference in weight among groups (*P* > 0.05). At 0 day, the weight in Hi MLA + taVNS and Hi saline + taVNS group after 21 days modeling were significantly lower than that in the control group (*P* < 0.001, *P* < 0.01). At 25 days, compared with control group, the weight in Hi MLA + taVNS and Hi saline + taVNS group significantly decreased (*P* < 0.01, *P* < 0.01) (as shown in Fig. 5a2).

### Changes in sucrose preference of SPT

Before the experimental procedures, there was no significant difference in sucrose preference of rats among groups (*P* > 0.05). When CUMS procedure or taVNS was involved at different time points, the sucrose preference of rats in each group changed significantly. Compared with the control group, the sucrose preference of rats in the other three groups decreased significantly at 0 day (*P* < 0.001). After taVNS intervention, this result was reversed. Compared with the model group, the sucrose preference of rats in the taVNS group increased significantly (*P* < 0.001) at 21 days. And the sucrose preference of rats in α7nAchR(−/−) + taVNS group decreased (*P* < 0.001) compared to that in taVNS group (as shown in Fig. 4b1).

At baseline (− 21 days), there was no significant difference in sucrose preference of rats among groups (*P* > 0.05). At 0 day, the sucrose preference in Hi MLA + taVNS and Hi saline + taVNS group after 21 days modeling were significantly lower than that in the control group (*P* < 0.01, *P* < 0.01). At 25 days, compared with Hi saline + taVNS group, the sucrose preference in Hi MLA + taVNS group significantly decreased (*P* < 0.05) (as shown in Fig. 5b2).

### Changes in horizontal and vertical movement scores of OFT

Similar to what was observed in SPT, there was no significant difference among groups in the scores of horizontal and vertical movement at − 21 days (*P* > 0.05). As shown in Fig. 4c1, d1, compared with the control group, the scores of horizontal and vertical movement of rats in the other three groups decreased significantly at 0 day (*P* < 0.001). Interestingly, taVNS intervention reversed this trend. Compared with the model group, the scores of horizontal and vertical movement in taVNS group increased significantly at 21 days (*P* < 0.001, *P* < 0.01, respectively). However, the scores of horizontal movement in α7nAchR(−/−) + taVNS group decreased comparing to that in taVNS group (*P* < 0.05).

At baseline (− 21 days), there was no significant difference among groups in the scores of horizontal and vertical movement (*P* > 0.05). At 0 day, the scores in Hi MLA + taVNS and Hi saline + taVNS group after 21 days modeling were significantly lower than that in the control group (score of horizontal movement, *P* < 0.01, *P* < 0.01; score of vertical movement, *P* < 0.01, *P* < 0.001). At 25 days, compared with Hi saline + taVNS group, the horizontal scores in Hi MLA + taVNS group significantly decreased (*P* < 0.05) (as shown in Fig. 5c3, d4).

### Changes in immobility time of FST

There was no significant difference among groups in the immobility time of FST at − 21 days (*P* > 0.05). After modeling (0 day), the immobility time in control group was significantly longer than that in the other three groups (*P* < 0.001), and FST indicated that model, taVNS and α7nAchR(−/−) + taVNS groups showed depressive-like behavior. After taVNS intervention for 21 days, compared with model group, the immobility time markedly decreased in taVNS group (*P* < 0.01). Compared with taVNS group, the immobility time in α7nAchR(−/−) + taVNS group has an increasing tendency, but there was no statistical difference (*P* > 0.05) (as shown in Fig. 4e1).

At baseline (− 21 days), there was no significant difference among groups in the immobility time of FST (*P* > 0.05). At 0 day, the immobility time in Hi MLA + taVNS and Hi saline + taVNS group after 21d modeling were significantly higher than that in the control group (*P* < 0.01, *P* < 0.01). At 25 days, compared with Hi saline + taVNS group, the immobility time in Hi MLA + taVNS group significantly increased (*P* < 0.01) (as shown in Fig. 5e2).

### Western blot analysis

#### Expression of α7nAchR in the hippocampus

**Fig. 6:**
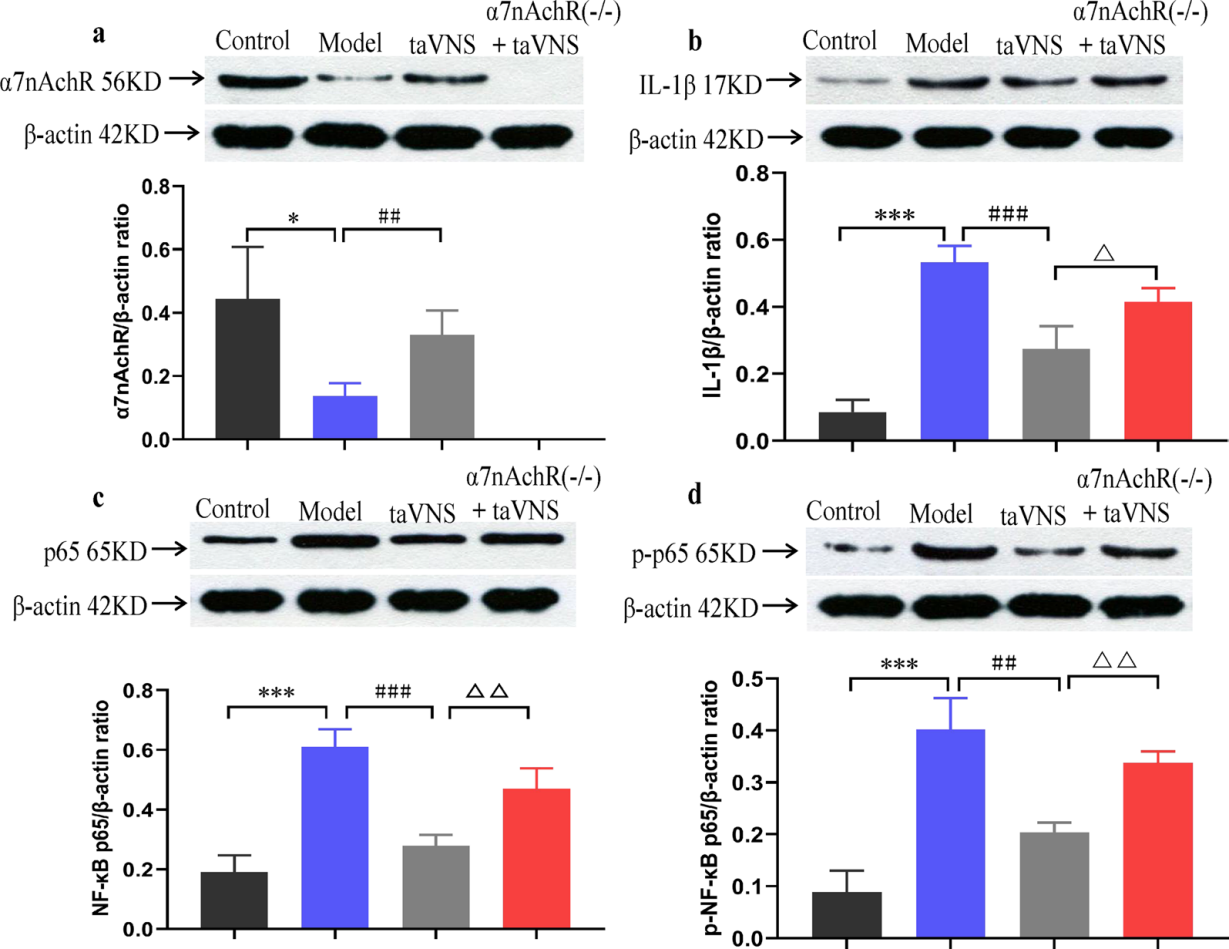
The effect of taVNS on the expression of α7nAchR, IL-1β, NF-kB p65 and p-NF-kB p65 in the hippocampus. **a** Effect of taVNS on the expression of α7nAchR in hippocampus of rats. **b** Effect of taVNS on the expression of IL-1β in hippocampus of rats. **c** Effect of taVNS on the expression of NF-kB p65 in hippocampus of rats. **d** Effect of taVNS on the expression of p-NF-kB p65 in hippocampus of rats. The values are presented as mean ± SD. One-way ANOVA with Tukey post hoc test was performed for statistical analysis. *P < 0.05 vs. the control group, ***P < 0.001 vs. the control group; ##*P* < 0.01 vs. the model group, ###*P* < 0.001 vs. the model group; △*P* < 0.05 vs. the taVNS group, △△*P* < 0.01 vs. the taVNS group (n = 6 per group)

There was a significant difference in α7nAchR expression among the groups. The expression of α7nAchR was significantly lower in the model group compared to that in the control group (*P* < 0.05). In comparison with the model group, the higher expression level of α7nAchR showed in taVNS group (*P* < 0.01). Because of α7nAchR knockout in α7nAchR(−/−) + taVNS group, α7nAchR protein was not expressed (Fig. 6a).

### Expression of IL-1β in the hippocampus

Compared with the control group, the expression of IL-1β in the model group was significantly up-regulated (*P* < 0.001). Of note, taVNS obviously decreased the expression of hippocampal IL-1β when compared with that in the model group (*P* < 0.001). However, the expression of hippocampal IL-1β in α7nAchR(−/−) + taVNS group was higher than that in taVNS group (*P* < 0.05) (Fig. 6b).

### Expression of NF-kB p65 and p-NF-kB p65 in the hippocampus

Compared with the control group, the expression of NF-kB p65 and p-NF-kB p65 in the model group increased significantly (*P* < 0.001). Importantly, taVNS reversed the upregulation of hippocampal NF-kB p65 and p-NF-kB p65 when compared with that in model group with statistical significance (*P* < 0.01, *P* < 0.001, respectively). Nevertheless, the expression of NF-κB p65 and p-NF-kB p65 increased in α7nAchR(−/−) + taVNS group compared with taVNS group (*P* < 0.01) (Fig. 6c, d).

### Immunofluorescence staining

#### The effect of taVNS on the expression of hippocampal microglia and α7nAchR

**Fig. 7:**
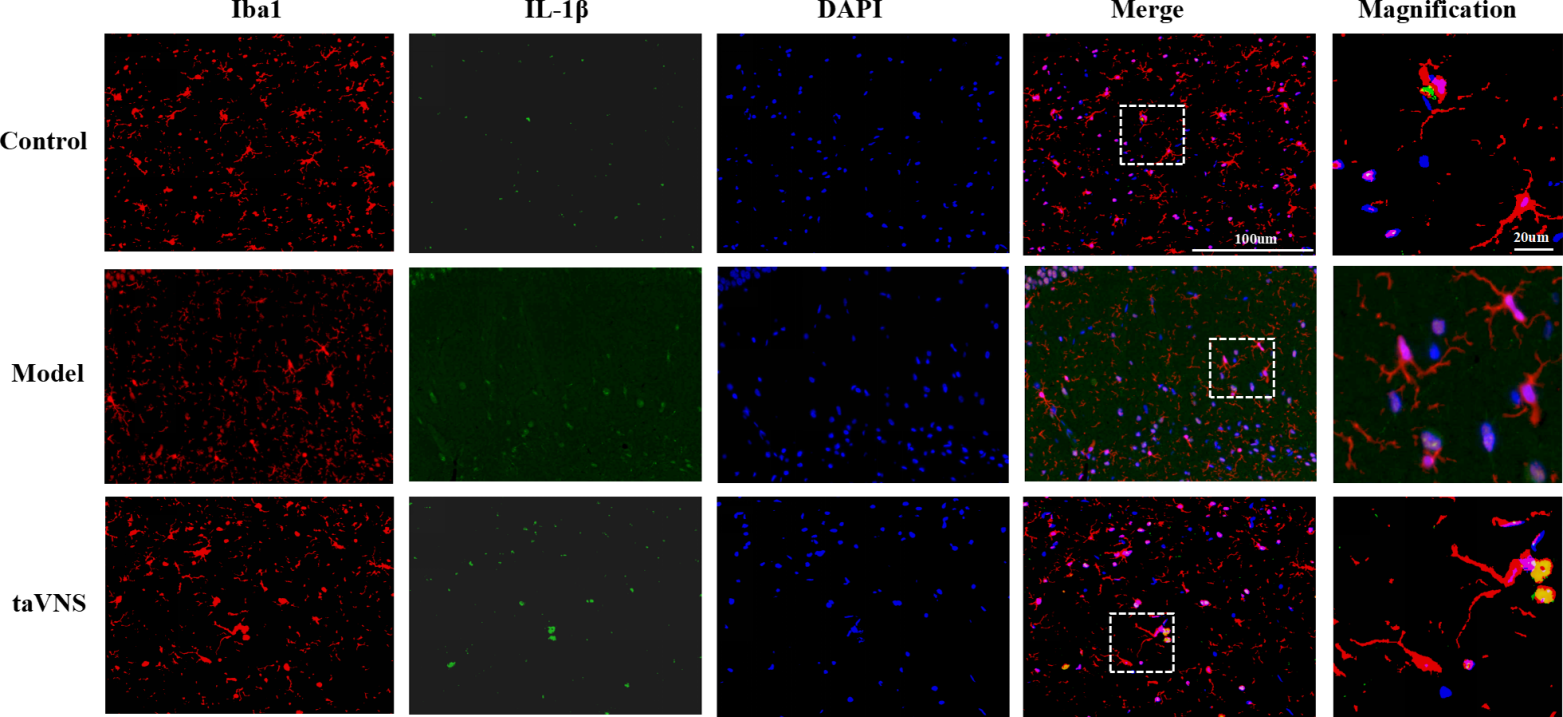
Immunofluorescence staining of hippocampal sections in each group rats (IL-1β) (*n* = 3, per group). Compared with control group and taVNS group, representative confocal images of brain sections shows more expression of IL-1β (green) in model group. The microglia transform from ramified morphology (control group) to ameboid form (model group) to ramified state (taVNS group). Compared with control group and taVNS group, the co-expression between pro-inflammatory cytokine IL-1β (green) and microglia (red) increased in model group, while the result was reversed in taVNS group. IL-1β (green), Iba-1 (red), DAPI (blue), Merge (yellow); 40 times objective lens, scale bar: 100 μm and 20 μm magnification

**Fig8:**
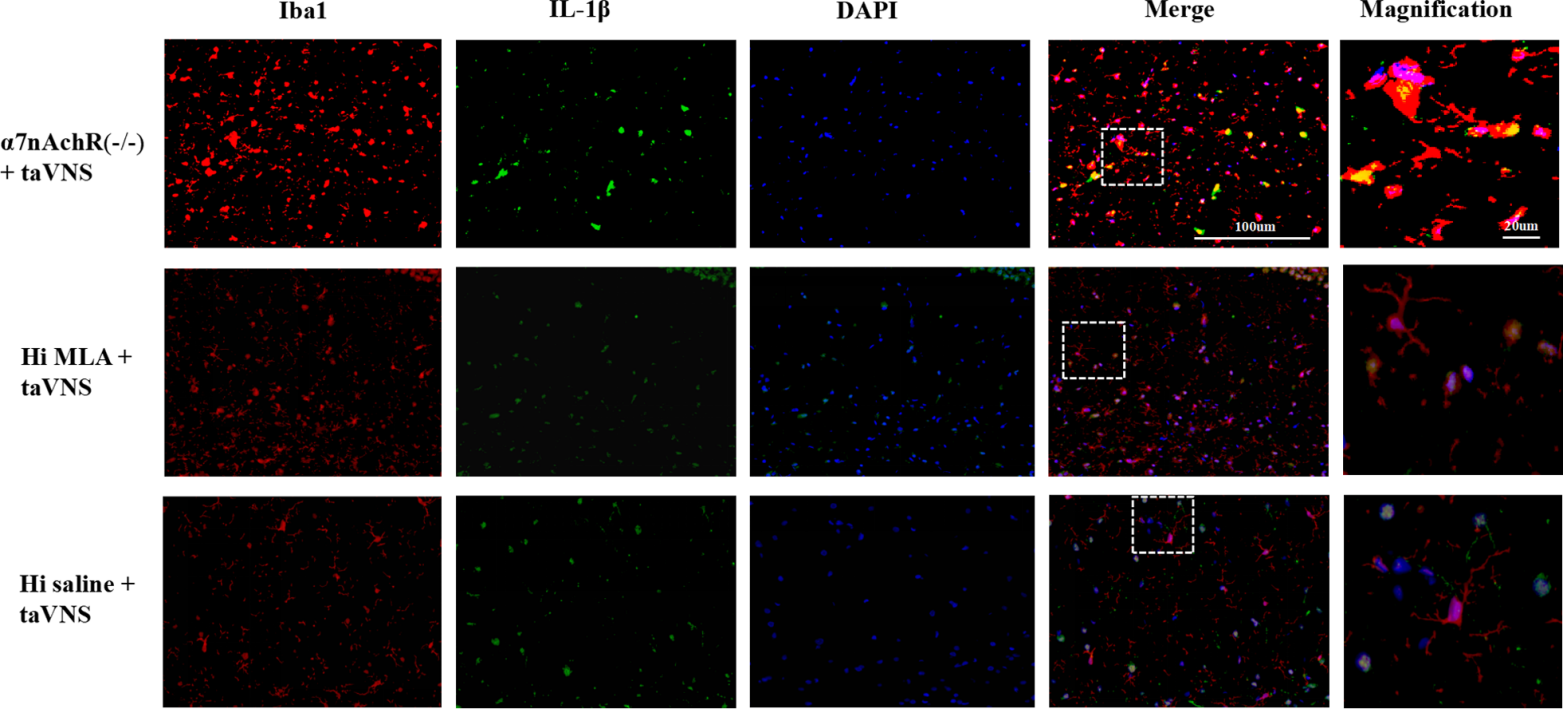
Immunofluorescence staining of hippocampal sections in each group rats (IL-1β) (*n* = 3, per group). The microglia transform from ameboid form (α7nAchR(−/−) + taVNS group and Hi MLA + taVNS group) to ramified state (Hi saline + taVNS). Compared with α7nAchR(−/−) + taVNS group and Hi MLA + taVNS group, the co-expression between pro-inflammatory cytokine IL-1β (green) and microglia (red) decreased in Hi saline + taVNS group. IL-1β (green), Iba-1 (red), DAPI (blue), Merge (yellow); 40 times objective lens, scale bar: 100 μm and 20 μm magnification

In this study, immunofluorescence staining of Iba-1 in the microglia was used to observe the morphology of microglia in the hippocampus of rats in each group, in order to determine the resting or activated state of microglia. The microglia was small and slender in control group, suggesting that it is in resting state. Compared with control group, microglia was activated in model group, which showed irregular enlargement of cell body. The morphology of microglia in taVNS group is not obviously activated when compared with model group. The morphology of microglia was amoeba-like activated with larger cell bodies and shorter protuberations in α7nAchR(−/−) + taVNS and Hi MLA + taVNS group than that in Hi saline + taVNS group. α7nAChR was mainly expressed on microglial membrane in control and taVNS group, while the co-expression between α7nAChR and microglia in in control and taVNS group was higher than that in model group. And Compared with α7nAchR(−/−) + taVNS group and Hi MLA + taVNS group, the co-expression between α7nAchR and microglia increased in Hi saline + taVNS group (Figs. 7, 8).

### The effect of taVNS on the expression of hippocampal microglia and IL-1β

**Fig. 9:**
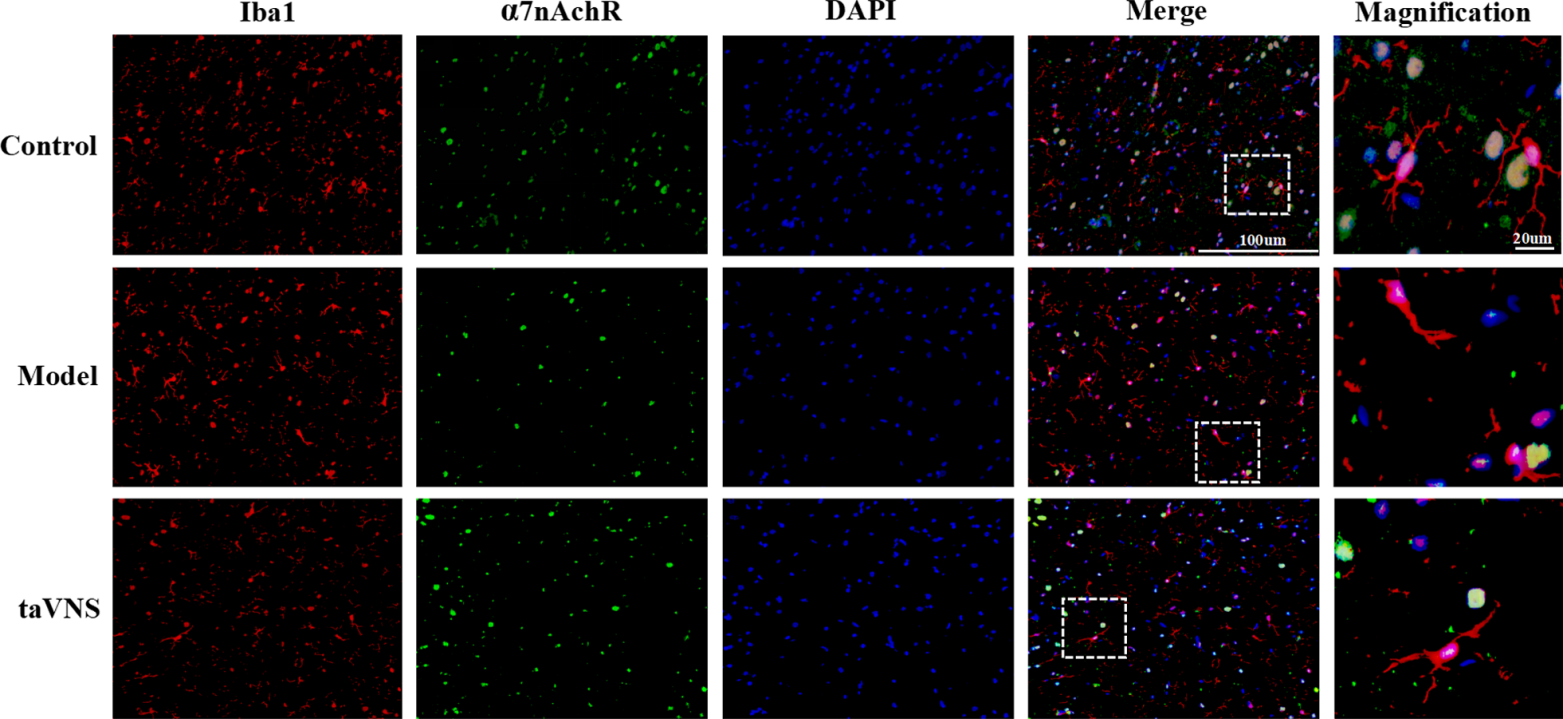
Immunofluorescence staining of hippocampal sections in each group rats (α7nAchR) (*n* = 3, per group). Compared with control group and taVNS group, representative confocal images of brain sections show less expression of α7nAchR (green) in model group. The microglia transform from ramified morphology (control group) to ameboid form (model group) to ramified state (taVNS group). Compared with control group and taVNS group, the co-expression between α7nAchR (green) and microglia (red) decreased in model group. α7nAchR (green), Iba-1 (red), DAPI (blue), Merge (yellow); 40 times objective lens, scale bar: 100 μm and 20 μm magnification

**Fig. 10:**
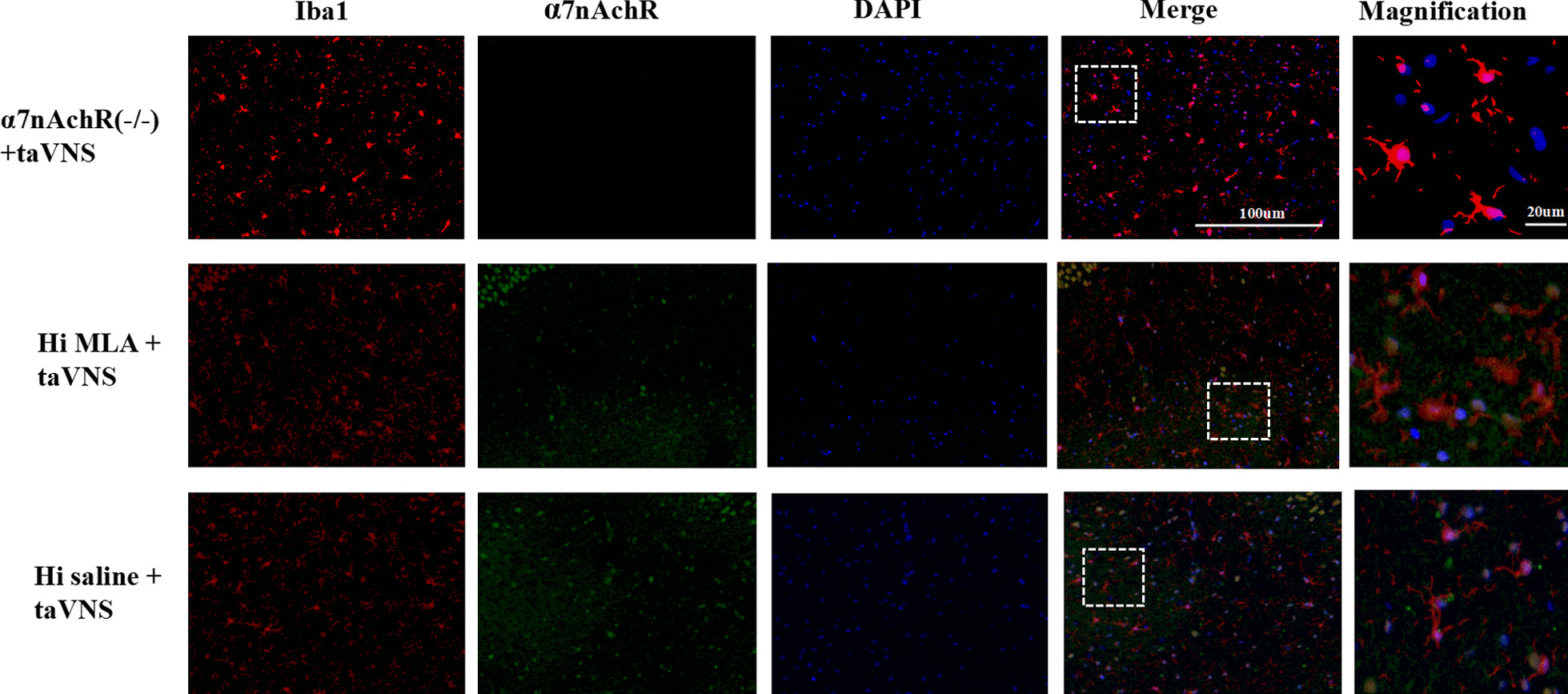
Immunofluorescence staining of hippocampal sections in each group rats (α7nAchR) (*n* = 3, per group). Compared with α7nAchR(−/−) + taVNS group and Hi MLA + taVNS group, representative confocal images of brain sections shows more expression of α7nAchR (green) in Hi saline + taVNS group. The microglia transform from ameboid form (α7nAchR(−/−) + taVNS group and Hi MLA + taVNS group) to ramified state (Hi saline + taVNS). Compared with α7nAchR(−/−) + taVNS group and Hi MLA + taVNS group, the co-expression between α7nAchR (green) and microglia (red) increased in Hi saline + taVNS group. α7nAchR (green), Iba-1 (red), DAPI (blue), Merge (yellow); 40 times objective lens, scale bar: 100 μm and 20 μm magnification

IL-1β has little or no expression in control group. The co-expression between pro-inflammatory cytokine IL-1β and microglia was significantly increased in model group, while the result was reversed in taVNS group. Compared with α7nAchR(−/−) + taVNS group and Hi MLA + taVNS group, the co-expression between pro-inflammatory cytokine IL-1β and microglia decreased in Hi saline + taVNS group. The morphological changes of microglia in each group were consistent with those described previously (Figs. 9, 10).

### The number of activated or resting state microglia

**Fig. 11:**
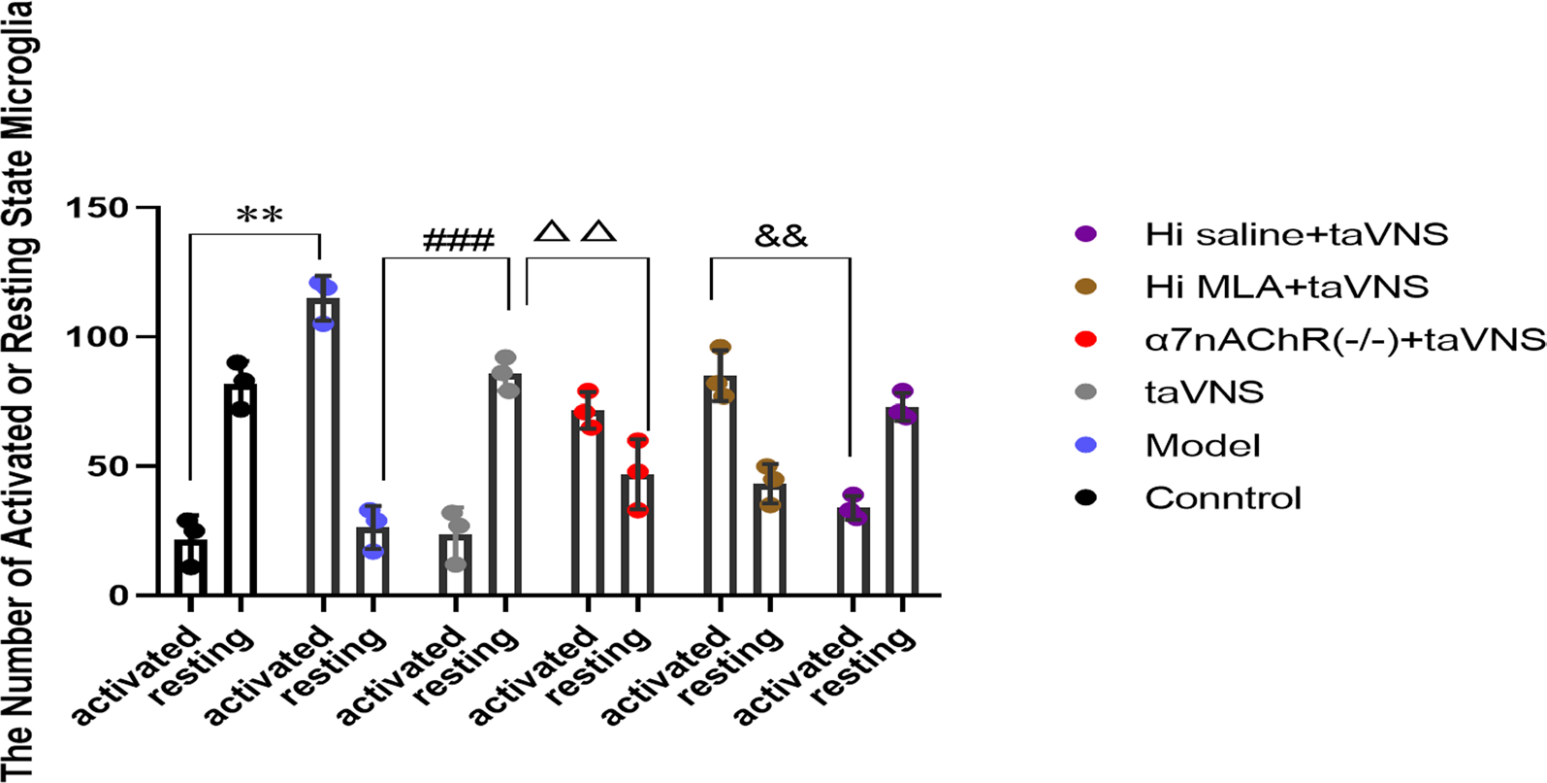
The number of activated or resting state microglia**.** **P < 0.01 vs. the control group; ###*P* < 0.001 vs. the model group; △△*P* < 0.01 vs. the taVNS group; &&*P* < 0.01 vs. the Hi MLA + taVNS group (*n* = 3 per group)

Compared with the control group, the number of activated microglia increased significantly in the model group (*P* < 0.01). And the number of resting microglia increased in the model group increased than that in the taVNS group (*P* < 0.001). Compared with the taVNS group, the number of resting microglia decreased significantly in the α7nAchR(−/−) + taVNS group (*P* < 0.01). And the number of activated microglia decreased in the Hi saline + taVNS group than that in the Hi MLA + taVNS group (*P* < 0.01) (Fig. 11).

## Discussion

Although the pathogenesis of depression is not clear, a large number of studies has shown that neuroinflammation caused by stress is closely related to depression [40, 41] Anti-inflammatory treatment has become a new option for antidepression, and recent meta-analyses have supported this concept [42]. Previous studies have shown that taVNS can effectively treat depression [43]. Based on the relationship between VN and immune system [44], we speculate that the regulation of neuroinflammation may play an important role in the antidepressant effect of taVNS. However, due to the lack of evidence and heterogeneity of methods, this conjecture is still controversial. Therefore, in this study, we established a rat depression model by CUMS method, which was proved to be parallel to the symptoms of depression and accurately summarized the human condition. We tried to provide some convincing findings on the antidepressant-like effect of taVNS at the molecular level and a new framework for exploring new treatments of depression.

Chronic and long-term stressful events in the life are main causes of depression in the population. CUMS, which is considered to be one of the most effective and reliable methods to establish a rat model of depression, generally lasts for more than 21 days in the modeling cycle [45]. This method simulates a variety of stressors of depression. Animals exposed to CUMS will show a series of depression-like behaviors such as behavioral despair, anhedonia, less exploration and less locomotion [46]. The methods for evaluating this behavior include OFT, SPT, and FST. In this study, we evaluated depressive-like behavior in CUMS model rats by using the three behavioral methods described above. We come to the conclusion that taVNS can alleviate the depression-like behaviors induced by CUMS in rats, which is to some extent consistent with our previous clinical studies [47]. It was found that CUMS significantly decreased the exploration and locomotion ability of rats in OFT, and decreased the immobility time as well as the percentage of sucrose preference in SPT. In contrast, the results in α7nAchR(−/−) rats did not improve after taVNS intervention. Thus, we speculate that α7nAchR is the target of taVNS to exert its central antidepressant effect.

**Fig. 12:**
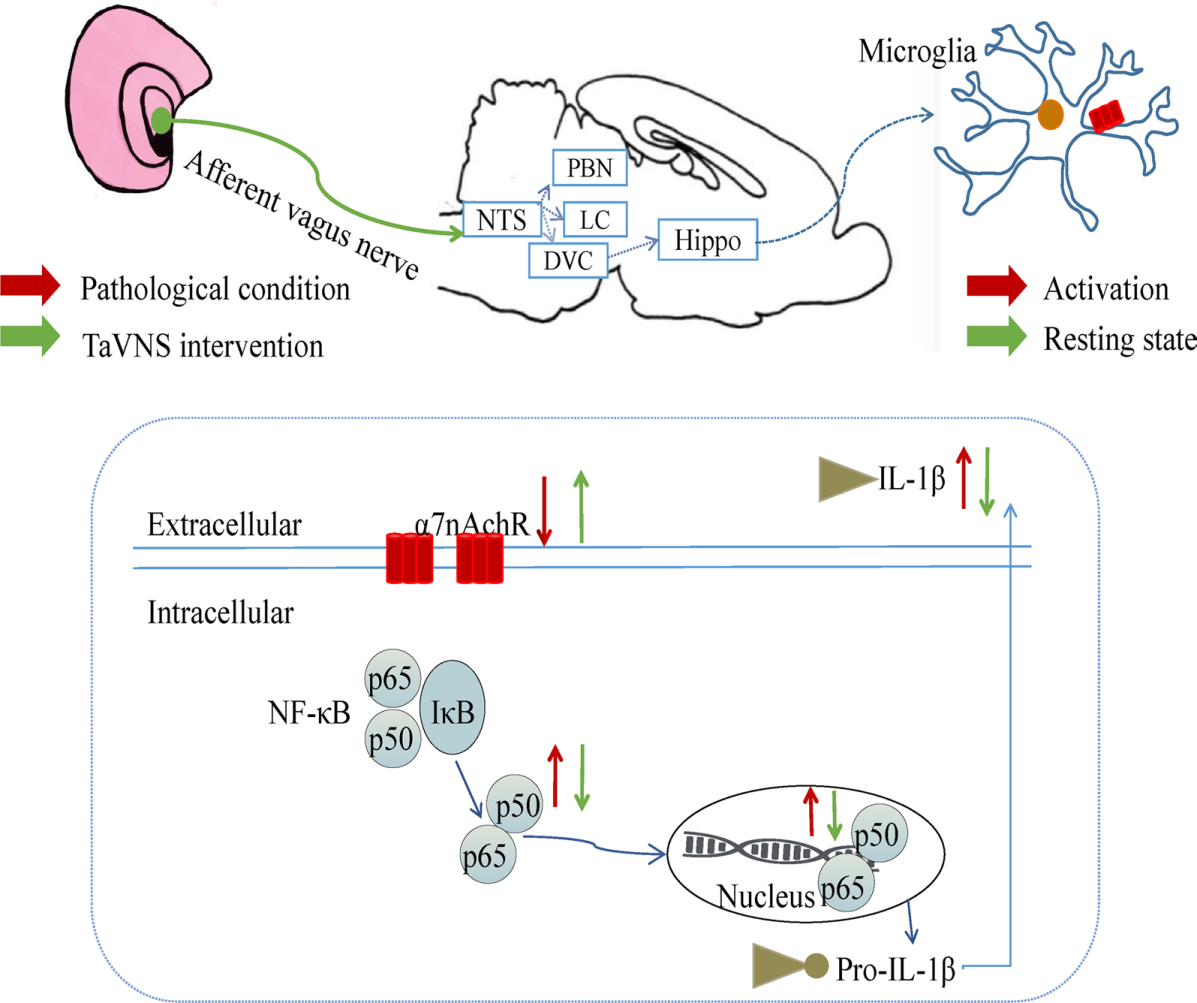
The antidepressant-like effect of taVNS may be related to the regulation of α7nAchR/NF-κB signal pathway. The α7nAChR is the pivotal receptor that mediates the inflammation reflex of the vagus nerve. TaVNS stimulates ABVN, and the brain receives information from the vagus afferent fibers. The afferent fibers project to the NTS in the brainstem, and then form indirect ascending projections from the NTS to hippocampus. TaVNS can affect hippocampus anti-inflammation mediated by α7nAChR on microglia. Hippocampal microglial cells at resting state do not exhibit activation for inflammatory signal pathway. This means that NF-κB signal pathway remains inactivate. However, in pathological condition, the degradation of IκB protein unmasks NF-κB from inactive to active state and the active NF-κB leads to nuclear translocation. NF-κB p65 occurs nuclear translocation to regulate the IL-1β

α7nAChR plays an essential role in the central anti-inflammation process that regulates microglia function in inflammation, and distributed widely in CNS, not only in neurons, but also in glial cells [46]. The hippocampus is a key nucleus involved in the regulation of cognition and emotion, where there are expressions of many nAChR subunits. But the most highly expressed receptor is α7nAChR [49]. Acting on α7nAchR can regulate synaptic plasticity of the hippocampus [50]. Microglial abnormalities have been implicated in a wide range of brain diseases, including depression [51]. Microglia exhibits a broad spectrum of activation states upon receiving various stimuli, and can be divided into the M1 phenotype that secrets the pro-inflammatory cytokines such as TNF-α, IL-1β, IL-6 and the M2 phenotype that produces the anti-inflammatory factors such as IL-4 and IL-10 [52]. It is widely known that the vagus nerve has positive anti-inflammatory actions of regulating the function of immune cells through activation of α7nAChR. The α7nAChR is the pivotal receptor that mediates the inflammation reflex of the vagus nerve [13, 53]. TaVNS stimulates ABVN, and the brain receives information from the vagus afferent fibers. The afferent fibers project to the nucleus tractus solitarius (NTS) and locus coeruleus (LC) in the brainstem, and then form direct and indirect ascending projections from the NTS to hypothalamus, amygdala, hippocampus, frontal lobe, and other areas of the brain [54, 55]. In addition, taVNS can affect cholinergic anti-inflammation mediated by α7nAChR on macrophage, microglia and neuron [56]. Our studies also clarified that the morphology of microglia changed from an amoebic-like activated state to a resting state with small cell bodies, slender protrusions and free stretching and the level of IL-1β secreted by the M1 phenotype decreased after taVNS intervention of 21 days. While the morphology of microglia in α7nAchR(−/−) rats was amoebic-like activation mostly, and the content of hippocampal IL-1β in these rats was higher than that in the taVNS group. In this experiment, we used the methyllycaconitine (MLA), which is a specific α7nAchR antagonist [33], and infused into the dorsal hippocampus to explore the role of α7nAchR in hippocampus. The result has shown that taVNS did not significant reversed the depression-like behavior in Hi MLA + taVNS group compared with Hi saline + taVNS group. Nicotine mediated by α7nAchR displayed an anti-inflammatory activity involving inhibition of NFκB-signaling. Along with observation, it was shown that the receptor responsible for this response was the α7nAChR subtype [23]. Microglial cells at resting state do not exhibit activation for inflammatory signal pathway reflecting in binding of inhibitor of κB (IκB) to NF-κB p65 and p50 subunits within cytoplasm. This means that NF-κB signal pathway remains inactivated [58]. However, in the circumstance of over-stress, IκB becomes phosphorylated and exposed to proteolytic degradation [59]. After that, the degradation of IκB protein unmasks NF-κB from inactive to active state and the active NF-κB leads to nuclear translocation. NF-κB p65 occurs nuclear translocation to regulate the pro-inflammatory cytokines, such as IL-1β [60]. NF-κB signal is a critical mediator of the anti-neurogenic and behavioral actions of stress. NF-κB is closely related to neuroinflammation. On one hand, NF-κB, as a multi-effect regulator, is involved in the regulation of inflammatory mediators and the transcription and expression of inflammatory cytokines; on the other hand, inflammatory factors such as IL-1β, can activate NF-κB. This cycle aggravates the inflammatory response [61]. Experiments have shown that stress inhibition of neurogenesis in the hippocampus, which has been demonstrated on the pro-depressive effects of stress, is blocked by an inhibitor of NF-κB [62]. Our studies also observed that the content of IL-1β and NF-κB p65 increased after CUMS modeling, but the phenomenon is alleviated by taVNS. Nevertheless, IL-1β, NF-κB p65 and p-NF-κB p65 were still high in α7nAchR(−/−) rats, even after the intervention of taVNS. Furthermore, the activation of α7nAChR blocked LPS-mediated NF-κB nuclear translocation, which indicated that the observed anti-inflammatory effect may be mediated through inhibition of the NF-κB pathway [63]. α7nAChR/NF-κB signal pathway is a crucial pathway to regulate CNS neuroinflammation [64], and the results of this experiment suggest that taVNS can up-regulate the expression of α7nAchR on hippocampal microglia. α7nAchR prevents the NF-κB nuclear translocation and the expression of phosphorylated-p65, thereby reducing the content of IL-1β. Therefore, the antidepressant effect of taVNS is related to hippocampal α7nAchR/NF-κB signal pathway (Fig. 12).

## Conclusion

This study shows that the taVNS alleviates depression-like behavior induced by CUMS in rats and is related to the α7nAchR-mediated neuroinflammation in the hippocampus. Importantly, our results show that taVNS intervention can effectively relieve depression-like behavior in CUMS model rats. This antidepressant effect is due to anti-inflammation by upregulation of the α7nAchR, which prevents the expression of NF-κB nuclear translocation and its phosphorylated-p65 to reduce the production of pro-inflammatory cytokines such as IL-1β. Therefore, the antidepressant-like effect of taVNS may be related to the regulation of α7nAchR/NF-κB signal pathway. We hope that this study may provide a new idea for exploring neuromodulation techniques in the treatment of depression from the perspective of anti-inflammation.

## Data Availability

All data generated or analyzed during this study are included in this published article.

## Abbreviations

α7nAchR: α7 Nicotinic acetylcholine receptor
CUMS: Chronic unpredictable mild stress
CNS: Central nerve system
FDA: Food and Drug Administration
FST: Force swimming test
IL-1β: Interleukin-1β
Hi: Hippocampus
LPS: Lipopolysaccharides
NTS: Nucleus of tract solitary
NF-κB p65: Nuclear factor κB p65
OFT: Open field test
p-NF-κB p65: Phosphorylated-nuclear factor κB p65
SPT: Sucrose preference test
TNF-α: Tumor necrosis factor-α
taVNS: Transcutaneous auricular vagus nerve
VNS: Vagus nerve stimulation
